# Regulatory Sandboxes: A Cure for mHealth Pilotitis?

**DOI:** 10.2196/21276

**Published:** 2020-09-15

**Authors:** Abhishek Bhatia, Rahul Matthan, Tarun Khanna, Satchit Balsari

**Affiliations:** 1 The Lakshmi Mittal and Family South Asia Institute Harvard University Cambridge, MA United States; 2 FXB Center for Health and Human Rights Harvard TH Chan School of Public Health Boston, MA United States; 3 The Takshashila Institution Bengaluru India; 4 Trilegal Bengaluru India; 5 Harvard Business School Boston, MA United States; 6 Department of Emergency Medicine Beth Israel Deaconess Medical Center and Harvard Medical School Boston, MA United States

**Keywords:** COVID-19, mHealth, digital health, design thinking, regulation, intervention, regulatory sandbox

## Abstract

Mobile health (mHealth) and related digital health interventions in the past decade have not always scaled globally as anticipated earlier despite large investments by governments and philanthropic foundations. The implementation of digital health tools has suffered from 2 limitations: (1) the interventions commonly ignore the “law of amplification” that states that technology is most likely to succeed when it seeks to augment and not alter human behavior; and (2) end-user needs and clinical gaps are often poorly understood while designing solutions, contributing to a substantial decrease in usage, referred to as the “law of attrition” in eHealth. The COVID-19 pandemic has addressed the first of the 2 problems—technology solutions, such as telemedicine, that were struggling to find traction are now closely aligned with health-seeking behavior. The second problem (poorly designed solutions) persists, as demonstrated by a plethora of poorly designed epidemic prediction tools and digital contact-tracing apps, which were deployed at scale, around the world, with little validation. The pandemic has accelerated the Indian state’s desire to build the nation’s digital health ecosystem. We call for the inclusion of regulatory sandboxes, as successfully done in the fintech sector, to provide a real-world testing environment for mHealth solutions before deploying them at scale.

## Introduction

Millions of dollars have been spent on digital health interventions in the global south in the past few years, often with the generous support of influential global philanthropic organizations [1]. Many have not had the anticipated impact at scale despite successful pilot studies [2,3]. The COVID-19 pandemic has amplified and accelerated this mobile health (mHealth) pilotitis with unvalidated and untested social distancing scoreboards, apps, and forecasting models proliferating around the world [4]. As communities and businesses struggle with the uncertainties associated with reopening society, a wide range of technological solutions are being proposed—including syndromic surveillance trackers, electronic passes controlling access to building and transport hubs, and contact-tracing apps and devices [5]. These unvetted tools risk detracting policymakers from focusing on the basics of pandemic response [6].

Over the past decade, the implementation of digital health tools has largely suffered from 2 limitations. First, technocentric interventions commonly ignore what Toyama refers to as the law of amplification in his book *Geek Heresy* [7]. Technology, he postulates, is most likely to succeed when it seeks to augment and not alter human behavior. Secondly, end-user needs are poorly understood in the interest of creating new markets, leading to a significant decrease in usage over time. This attrition is seldom studied but is important while evaluating digital health implementation, as described by Eysenbach [8].

The minimum viable product—the norm in the start-up industry—is anathema in medicine and public health. Speed need not supplant rigor. In this article, we propose a measured iterative approach to developing, validating, and scaling digital health interventions, using illustrative examples from India.

### The Law of Amplification

Before the pandemic, there were over already 350,000 apps in the mHealth category across app stores, though few have had any longevity [9]. Most have relied on misguided assumptions of how humans consume and interact with technology and provided false reassurances to users and policymakers [10]. Those that have been somewhat successful and had impact at scale have demonstrated a commitment to understanding the context and have adopted key design thinking principles, including an iterative and inclusive development process [11,12]. They recognize Toyama’s law of amplification [7] that states that technology by itself will not fundamentally change behavior; it will merely amplify it.

The motion picture, radio, television, and finally, massive open online courses (MOOCs) were all expected to fundamentally change how we educated students. They did not [13-15]. While the internet substantially eased the pursuit of knowledge, it had, for the most part, augmented but not entirely replaced classrooms—until the pandemic fundamentally modified human behavior. How we seek education and health has now changed; human mobility is restricted, and because students cannot travel to campuses, we are witnessing an explosive growth in online learning tools as technology responds to amplify human behavior [16].

Telemedicine, which was expected to substantially improve access to general and specialized care, has also not scaled as anticipated. Legal, financial, behavioral, and infrastructural limitations were hard to overcome in both high- and low-income settings [17]. The pandemic has, however, disrupted primary and chronic care globally with particularly devastating consequences in resource-poor settings. In the United States, for example, this public health emergency has accelerated negotiations about regulation and remuneration in telemedicine that had otherwise stalled for years [18]. The 3-month-long nationwide lockdown in India precluded hundreds of thousands of patients from receiving routine home care by community health workers or periodic checks, for example, by their endocrinologists and oncologists [19]. While physicians in the developing world have routinely been using phone-based apps, especially WhatsApp, to communicate with patients [20], the clinical and financial imperatives posed by the pandemic prompted many hospitals, both private and public, to adopt telemedicine solutions expeditiously [21]. The government of India released Telemedicine Practice Guidelines on March 25, 2020, allowing teleconsultation for essential non-COVID services, further embedding telemedicine in routine health care delivery [22].

In contrast, COVID-19 mobility dashboards and contact-tracing apps promoted by governments, technology companies, consulting companies, and academics largely failed to alter the course of the epidemic [23]. Decision rooms of health departments were inundated with presentations of prediction models, despite the unavailability of data required to parametrize these models adequately [24]. Policymakers are weighed down by the low signal-to-noise ratio in the data generated by the health system along with the digital solutions presented to them, compelling the WHO director-general to label the phenomenon an “infodemic” [25,26]. These solutions could not amplify what did not exist—the capacity to test, isolate, or treat patients or to provide for citizens to safely stay home [27,28].

### The Tyranny of “Tablets”

The discordance between what is needed and what is supplied by the digital health tool ecosystem is not new. A closer look at the national programs digitizing their health data ecosystem in the countries with low- and middle-income reveals common patterns: overburdened healthcare providers enter large volumes of data into tablet computers or web apps in survey forms riddled with radio buttons, small fonts, and hard-to-navigate dropdown menus that are all anachronistic [29]. These tools reflect regulatory reporting and operational requirements originally designed for an analog world. In India, for example, individual programs must collect demographic data de novo, instead of relying on an application programming interface (API)-enabled import from an interoperable sister program. These data are then transferred with varying compliance and quality to dashboards with scant detail on how the summary statistics are calculated [30]. There is little evidence to show that these efforts result in timely action. Throughout, there is a greater emphasis on programmatic needs; and little attention is paid to the needs of the key users, the patient, and the health care provider—leading to user attrition over time [8,31].

### Sandboxing: The Need for Rapid Iteration

Governments currently find themselves facing tough choices between moving swiftly to reassure and protect populations by adopting promising though unvalidated technologies and proceeding cautiously, awaiting robust evidence, while the contagion spreads unabated [32]. We believe it is possible to strike a responsible balance. In 2019, the government of India published its sweeping vision for a digital health ecosystem for India’s 1.3 billion residents, the National Digital Health Blueprint (NDHB) [33]. The NDHB called for the provision of a regulatory sandbox—a controlled testing environment within which existing regulations may be temporarily relaxed to allow experimentation [34]. In August 2020, the Prime Minister announced the National Digital Health Mission (NDHM), and the National Health Authority (NHA) released its “Draft Health Data Management Policy” for public comment. Simultaneously, the NHA has launched the NDHM Sandbox, allowing integration and validation of third-party software by partnering with NDHM APIs [35]. We believe that these regulatory sandboxes can be the pill needed to combat the bane of mHealth pilotitis*,* in India and around the world. A sandbox will provide the enabling conditions administrators need to test new solutions in subsets of populations before mandating change at scale. They offer an opportunity to test what are often minimum viable digital health products in a responsible, controlled, and monitored real-world environment.

Let us consider, for example, follow-up home visits that are required of India’s army of accredited social health activists, or ASHAs (which means “hope” in Hindi). Several population health programs require ASHA workers to go door to door to inquire about the health status of children or expectant mothers or missed follow-up appointments. They then trudge back to the primary care centers to upload these data online, when tablet computers and broadband connectivity are not available in remote regions, and thousands of person-hours are lost weekly in this exercise. The (not entirely unfounded) fear of new technologies failing precludes administrators from experimenting or challenging age-old, resource-intensive, and sometimes ineffective practices. A sandbox would, in this case, provide state administrators with the regulatory flexibility needed to temporarily replace home visits with alternative digital solutions. In the context of the COVID-19 pandemic, there is a behavioral impetus to align the ASHA workers’ incentives to use digital solutions with patients’ needs. It is unsafe for ASHA workers to go door to door during a pandemic. The time saved by operating from home will allow them to either attend to increased household responsibilities resulting from shelter-in-place orders, spend more time with each patient, or call more patients. Patients benefit from access to care despite quarantining orders. A sandbox approach would allow the ASHA workers (and policy makers) to vet multiple solutions simultaneously, say, by comparing response rates among groups of patients self-isolating at home that received either push-notifications (inexpensive), or automated interactive text messaging (with minimal upfront development costs), or phone calls (resource-intensive). What may be effective in a certain demographic may not necessarily be generalizable, warranting constant feedback loops and the ability to rapidly iterate [36].

Such prototyping must begin upstream, incorporating end-user feedback early in the design process, as was successfully evinced during the development of a digital disease surveillance platform at a mass gathering in Maharashtra, India, in 2015. Preparatory consultation workshops with medical officers seconded to serve in pop-up clinics at the 2015 Kumbh Mela resulted in substantial data minimization and workflow acceleration while improving record-keeping [37].

### Agility and Caution

We recognize that regulatory sandboxes are at risk for abuse and will lose public trust if they seek to circumvent the nation’s laws on data protection and privacy [38]. The sandboxes could, however, themselves serve as powerful tools to test proposed technological solutions against India’s evolving data privacy jurisprudence and societal norms. Over the decades, the Indian state has amassed large volumes of health data; these data can be used to accelerate medical science and positively influence population health [39,40], but they can also result in discrimination and harm [41,42]. The ubiquity of mobile devices and cloud-based services will influence a society’s willingness to share their data—as will a better understanding of the harms and benefits of the exchange of health data. The sandboxes provide an opportunity to examine the terms on which society will engage with governments and private players to generate, exchange, and use health data and address fundamental questions about health data that are yet not fully answered across different contexts. For example, are data better utilized for research if they are secured at source and queried remotely with a role-based access, as with the Research Patient Data Registry (RPDR) by Partners Healthcare in Massachusetts [43]; or if datasets are exported for a fee with time and purpose limitations as with research data from the Centers for Medicare and Medicaid Services (CMS) database in the United States [44]; or without a fee, as with the MIMIC-III (Medical Information Mart for Intensive Care III) database, a comprehensive dataset of 12 years of deidentified health data from critical care units of Harvard’s Beth Israel Deaconess Medical Center [45]? Which of these approaches are best suited to the range of infrastructure and capability differences within a country?

Policymakers have neither the requisite evidence to evaluate the veracity of the promises made by digital health technologies nor the tools to allow rapid iteration during large-scale adoption [3,46]. We believe that the proposed sandboxes can provide an alternative model. These testbeds provide a means to reject what does not work in the communities served, to optimize interventions by tweaking them in a real-world context, and to generate the evidence required to scale up. Regulated with expertise, caution, and consent, they can serve as incubators for digital health innovation. Prior to making unvetted digital technologies a critical pillar of the COVID-19 response, or of the national public health system at large, a controlled launch in willing communities would provide critical insights on the technological feasibility, epidemiological utility, data protection capability, and society’s response to these innovations (Textbox 1).

**Objectives of regulatory sandboxes for mHealth.**

- Generate evidence for digital health interventions in the real-world context at reasonable scale
- Allow cautious relaxation of select regulations to allow for innovation
- Measure societal response to the proposed regulatory changes (and to the introduced innovations)
- Eliminate (early) interventions that fail to demonstrate impact or engender trust

### Will Sandboxing Work?

The idea of testing policy changes before enforcing them is not new. The economic reforms in China from 1979 to 2012, for example, were a result of several decentralized economic experiments across cities and provinces, where hard evidence and technological expertise were relied upon to inform the rapidly changing national economic policy [47,48]. The regulatory sandboxes described in the NDHB have more recent precedents in the fintech industry. First introduced in the United Kingdom in December 2015, sandboxes have rapidly spread across the Asia Pacific and used in the insurance, payments, and capital market sectors [34,49]. In November 2019, India’s 4 financial service regulators invited fintech companies to test their applications in regulatory sandboxes designed to cater to the banking, insurance, securities, and pensions sectors [47,50]. An mHealth regulatory sandbox could similarly catalyze innovation and infuse rigor in digital health implementation by providing a mechanism for evaluating the scientific validity, health impact, contextual relevance, regulatory compliance, and long-term feasibility of applications, prior to scale-up (Textbox 2).

**Evaluation framework for mHealth tools in regulatory sandboxes.**

Validation

- Does the innovation do what it says it does?
- Example claim: “Bluetooth technology will help identify most exposures among persons carrying enabled devices.”
- Validating this claim would require widespread testing in a target population to calculate the sensitivity and specificity of the contact-tracing app and to consequently alter detection parameters before a large-scale rollout.

Impact

- Does it result in improved clinical or population health outcomes?
- Example claim: “Human mobility dashboards predicting the impact of social distancing measures will help inform containment strategies.”
- Impact would need to be shown by a measurable change in the incidence of cases or by comparing outcomes with control group populations after implementing interventions based on information provided by the dashboard. Statistically significant testing strategies would be a prerequisite [51].

Relevance

- Are the improved outcomes a priority for the community?
- Example claim: “Digitizing vertical service delivery programs improves care quality.”
- Improved record keeping after digitization may not have an impact on population health if chronic inventory shortages and absenteeism are not addressed.

Compliance

- Do regulatory relaxations result in harm, now or later?
- Example claim: “It is necessary to collect location data to monitor individual movement during the pandemic.”
- In compliance with data protection laws, data should be aggregated or anonymized to the lowest possible resolution that allows epidemiologically sound interventions without risking individual or group reidentification or harm.

Feasibility

- Is it easy to use? Does it integrate with existing workflows?
- Example claim: “Digitization of discharge summary notes at the bedside expedites claims of reimbursements.”
- Feasible interventions should integrate into existing systems without introducing bottlenecks or resulting in flow reorganization with stakeholder buy-in. Given the volume of patients seen in the outpatient departments at public hospitals (several hundreds), this requirement, for example, may be debilitating in certain contexts. Providers should have the option of completing discharge notes within a reasonable timeframe.

Sustainability

- Can it scale to meet increased demand?
- Example claim: “A successful locally developed, locally deployed electronic medical record system is ready for national expansion.”
- Sustainable scale-up proposals would require assessing the flexibility of design features to accommodate for the heterogeneity in workflow, digital literacy, and ability to report needs across jurisdictions as well as the adequacy of decision support at scale. Early findings may signal the need for subsequent RCTs.

### Conclusion

The COVID-19 pandemic addresses 1 of the 2 key barriers to successful mHealth adoption—the discordance between the proposed solution and underlying human behavior is reduced as technological solutions are now better aligned with needs. We believe regulatory sandboxes address the other—the need for validation. As the National Health Authority and the Ministry of Health and Family Welfare launch the NDHM in August 2020, incorporating sandboxes is opportune and important [52]. Before prescribing solutions at a national scale, the sandboxes must examine what is permissible under existing laws, where existing laws fall short, and where additional enabling regulations are needed to advance clinical or population health.

The pandemic warrants urgent intervention, not *any* intervention. In heterogenous populations with large socioeconomic, cultural, and behavioral diversity, this approach is far more effective than ideological adherence. Medicine is after all the original home of randomized controlled trials—now the darling of economists [53]. The profession has, for decades, strived to make the practice evidence-based. It is time to apply the same vigilance to digital health implementation, even during the pandemic. Regulatory sandboxes may present an effective mechanism to do so.

## Abbreviations

ASHA: accredited social health activist
API: application programming interface
CMS: Centers for Medicare and Medicaid Services
mHealth: mobile health
MIMIC-III: Medical Information Mart for Intensive Care III
MOOC: massive open online course
NDHB: National Digital Health Blueprint
NDHM: National Digital Health Mission
NHA: National Health Authority
RPDR: Research Patient Data Registry
